# Transcriptomic Analysis Provides Insights into Grafting Union Development in Pecan (*Carya illinoinensis*)

**DOI:** 10.3390/genes9020071

**Published:** 2018-02-01

**Authors:** Zhenghai Mo, Gang Feng, Wenchuan Su, Zhuangzhuang Liu, Fangren Peng

**Affiliations:** 1 College of Forestry, Nanjing Forestry University, Nanjing 210037, China; m15895892025@163.com (Z.M.); fenggangpecan@163.com (G.F.); swcsdau@163.com (W.S.); zzliu91@163.com (Z.L.); 2 Co-Innovation Center for the Sustainable Forestry in Southern China, Nanjing Forestry University, Nanjing 210037, China

**Keywords:** grafting, pecan, transcriptome, graft union, hormone

## Abstract

Pecan (*Carya illinoinensis*), as a popular nut tree, has been widely planted in China in recent years. Grafting is an important technique for its cultivation. For a successful grafting, graft union development generally involves the formation of callus and vascular bundles at the graft union. To explore the molecular mechanism of graft union development, we applied high throughput RNA sequencing to investigate the transcriptomic profiles of graft union at four timepoints (0 days, 8 days, 15 days, and 30 days) during the pecan grafting process. After de novo assembly, 83,693 unigenes were obtained, and 40,069 of them were annotated. A total of 12,180 differentially expressed genes were identified between by grafting. Genes involved in hormone signaling, cell proliferation, xylem differentiation, cell elongation, secondary cell wall deposition, programmed cell death, and reactive oxygen species (ROS) scavenging showed significant differential expression during the graft union developmental process. In addition, we found that the content of auxin, cytokinin, and gibberellin were accumulated at the graft unions during the grafting process. These results will aid in our understanding of successful grafting in the future.

## 1. Introduction

Pecan (*Carya illinoinensis*), a member of the Juglandaceae family, is an economically important nut tree native to North America. It was introduced to China more than 100 years ago; however, for a long time, there was little incentive for its commercial planting, due to an extremely long juvenile stage, with approximately 10 years to maturity. Grafting is an effective approach to shorten the duration of vegetative growth, by which, pecan can start to bear fruits within 5–8 years. For the trees in the Juglandaceae family, grafting is more difficult in comparison to other fruit trees. In recent years, patch budding, one of the most commonly used grafting methods, conducted from July to September in China, has achieved about 90% of grafting success [1], which makes the large-scale cultivation of pecan possible. An in-depth understanding of the mechanism underlying successful grafting will help increase the production efficiency of pecan, as well as other trees in the future.

When grafting is performed, the grafted partners, scion, and rootstock are cut and joined together. Once the scion and rootstock come into intimate contact, an intricate structural and biochemical response occurs at the graft union for a successful graft. For woody trees, following the initial adhesion of grafted partners, the graft union undergoes two essential developmental processes: the formation of callus tissues, and the sufficient connection of functional vascular bundles between the scion and rootstock [2,3]. Therefore, graft union development is a process that involves cell division and differentiation at the graft junction.

Currently, reports regarding the molecular mechanism of graft union development are still limited. A cDNA-AFLP method was applied to investigate the gene expression in the graft process of hickory, and the research obtained 49 differentially expressed genes that were related to signal transduction, auxin transportation, metabolism, cell cycle, wound response, and cell wall synthesis [4]. In the hypocotyl grafts of *Arabidopsis*, changes in global gene expression were evaluated 24 h after grafting, and graft union development was revealed to involve signal transduction as well as cellular debris elimination [5]. In grapevine autografts, transcriptional changes were examined via whole genome microarray analysis, and the results revealed that graft union development triggered numerous gene expression changes related to wounding, cell wall modification, hormone signaling, and secondary metabolism [6]. A comparison the gene expression between the hetero- and autografts of grapevine indicated that genes involved in stress responses were up-regulated [7]. In recent years, RNA sequencing (RNA-seq) is a rapidly emerging transcriptome technology that can be performed without a reference genome. It has been employed to analyze the expression of mRNA and miRNA in hickory graft process, through which candidate genes involved in the auxin and cytokinin signaling were identified [8]; otherwise, a total of 12 candidate grafting-responsive miRNA were detected [9]. A comparative proteomic analysis of the hickory graft unions revealed that key enzymes involved in flavonoid biosynthesis were up-regulated 7 days after grafting [10].

Previously, we have paid attention to the morphological and proteomic changes in pecan homografts [11]. However, to the best of our knowledge, there have still been no reports describing the genes and gene networks underlying graft union development of pecan. In this study, we applied RNA-seq technology to construct mRNA libraries from the graft unions that were collected at 0, 8, 15, and 30 days after grafting, and analyzed the transcriptomic changes across the graft process.

## 2. Materials and Methods

### 2.1. Plant Material and Grafting Procedures

Pecan homografts were made in August using patch budding at the experimental orchard of Nanjing Forestry University (China). Graft unions (approximately 3 cm in length, the budding segment that includes the tissues of scion and the developing xylem of rootstock) were collected at 0, 8, 15, and 30 days after grafting, and immediately frozen in liquid nitrogen. The sampling timepoints were determined according to our histological analysis of the graft union developmental process in pecan homografts. In detail, the samples at 8 days, 15 days, and 30 days were selected for exploring the differentially expressed genes involved in the initial callus proliferation, massive callus proliferation accompanied by cambium establishment, and functional vascular bundles formation, respectively. Samples at 0 days were graft unions collected immediately from the scion and rootstock before grafting, and were used as controls. Three biological replicates were performed for each timepoint.

### 2.2. RNA Extraction, Library Construction, and Sequencing

Total RNA was isolated from the graft unions using the Universal Plant RNA Kit (BioTeke, Beijing, China) and treated with RNase-free DNase I (Takara) to degrade genomic DNA. RNA quality and quantity were monitored by a Nanodrop 1000 Spectrophotometer (Thermo Fisher Scientific, Wilmington, DE, USA) and Agilent 2100 Bioanalyzer (Agilent Technologies, Santa Clara, CA, USA). For each sample, about 3 μg of the total RNA that passed the quality examinations was used to prepare the cDNA library. Construction of sequencing libraries was performed by NEBNext^®^ UltraTM RNA Library Prep Kit for Illumina^®^ (NEB, Ipswich, MA, USA) according to the protocol. Briefly, the mRNA was enriched by oligo (dT)-attached magnetic beads and fragmented into short pieces, which were taken as templates for the first-strand and second-strand cDNA synthesis. Then, exonuclease/polymerase was used to convert the remaining overhangs into blunt ends. The resulting fragments were end-repaired by inserting an “A” base to the 3’ ends of the cDNA. NEBNext adapters with a hairpin loop structure were then ligated to the fragments. The library fragments were purified by an AMPure XP system (Beckman Coulter, Beverly, MA, USA) to select suitable cDNA fragments. Then, the products were amplified by PCR to create sequencing libraries. The constructed libraries were sequenced by an Illumina HiSeqTM 4000 platform (Biomarker Technology Company, Beijing, China). The sequencing raw data was deposited in the NCBI Sequence Read Archive (SRA) with the accession number SRP118757.

### 2.3. De Novo Assembly and Functional Annotation

After RNA sequencing, adapter sequences, poly-N reads, and low-quality reads from raw data were removed by in-house perl scripts to obtain clean reads. The resulting clean reads from all the samples were pooled for generating reference genes as far as possible. Trinity software with a k-mer length of 25 and other default parameters were used in the subsequent *de novo* assembly of transcriptome. Clean reads were assembled into contigs, and then further linked into transcripts through pair-end joining. The produced transcripts were clustered with a TGI clustering tool, and the longest transcripts were recognized as unigenes. For functional annotation, unigenes were compared against the following databases including NCBI non-redundant protein (Nr), Clusters of Orthologous Groups of proteins (COG), euKaryotic Orthologous Groups (KOG), Gene Ontology (GO), Kyoto Encyclopedia of Genes and Genomes (KEGG), Protein family (Pfam) and Swiss-Prot using the BLASTX program with E-value of 10^−5^.

### 2.4. Analysis of Differentially Expressed Genes (DEGs)

The clean reads sequenced from each sample were mapped back to the unigene library to calculate the abundance of unigenes. To quantify the gene expression level, FPKM (fragments per kilobase of exon per million mapped reads) was calculated in each sample by RSEM. Differential expression analysis was then performed using the DESeq R package for three comparisons (8 days vs. 0 days, 15 days vs. 0 days, and 30 days vs. 0 days). The false discovery rate (FDR) was applied to identify the *p* value threshold in multiple test and analysis. Only genes with FDR < 0.01 and more than two-fold change in expression between samples were considered as DEGs. GO enrichment analysis of DEGs was carried out by the topGO R package based on the hypergeometric test. Additionally, we used KOBAS software to test the enriched pathway of DEGs. GO terms and KEGG pathways with corrected *p* value ≤ 0.01 were recognized as significantly over-represented.

### 2.5. Validation of RNA-Seq Data by Quantitative Real-Time PCR (qRT-PCR)

RNA preparation with three biological replicates for each sample was conducted as described above. The first-strand cDNA synthesis was performed using a Prime-Script™ II First Strand cDNA synthesis kit (Takara Bio, Dalian, China) according to the manufacturer’s instructions. The primer sets for each unigene were designed by Primer Premier 5.0, and their sequences are listed in Table S1. qRT-PCR was carried out on an ABI 7500 Real-Time PCR System (Thermo Fisher Scientific, Inc., Waltham, MA, USA) with SYBR Premix Ex Taq™ II kit (Takara). Expression was calculated as 2^−ΔΔCt^ and normalized to that of the reference gene Actin.

### 2.6. Detection of Hormones Content by ELISA

Samples were taken from the graft union at 0, 8, 15, and 30 days after grafting with three biological replicates. The contents of endogenous indole-3-acetic acid (IAA), zeatin riboside (ZR), and gibberellin (GA) were measured with the enzyme linked immunosorbent assay (ELISA). The hormone ELISA kits were developed from China Agricultural University, which have been validated with GC-MS and HPLC method. The determination of hormone content was performed as outlined by [12].

## 3. Results and Discussion

### 3.1. De Novo Assembly and Functional Annotation

To gain a comprehensive overview of transcriptome associated with graft union development in pecan, samples at different time points (0 days, 8 days, 15 days, and 30 days after grafting) with three biological replicates were subjected to illuminate sequencing. Raw reads were cleaned to generate a total of 312.08 million high-quality reads, encompassing 93.22 gigabase pairs with an average GC percentage of 46.41%. As a whole, all libraries showed good sequencing quality with Q30 (sequencing error rate less than 0.1%) more than 86.58% (Table S2). After sequence cleaning, reads from all samples were mixed to perform *de novo* assembly by Trinity software. Short reads were assembled into 140,455 transcripts with N50 length of 1905 bp (50% of total assembly length is contained within unigenes at least 1905 bp) and an average length of 1178 bp. There were 31,127 (22.16%) transcripts in the range between 1000 bp to 2000 bp, and 25,188 (17.93%) with length more than 2000 bp. All transcripts were subsequently clustered to yield 83,693 unigenes with an N50 length of 1350 bp and mean length of 892 bp. Among these, 13,698 (16.37%) unigenes were in the range of 1000–2000 bp, and 8364 (9.99%) exceeded 2000 bp (Table 1).

All the 83,693 unigenes were aligned with available protein databases using the BLASTx algorithm with E-value of 10^−5^. The results showed that there were 11,762 (14.05%) unigenes matched in the COG database, 23,260 (27.79%) in the GO database, 13,859 (16.56%) in the KEGG database, 21,612 (25.82%) in the KOG database, 25,909 (30.96%) in the Pfam database, 23,243 (27.77%) in the Swiss-Prot database, and 38,793 (46.35%) in the NR database. In total, there were 40,069 unigenes annotated in at least one database, accounting for 47.88% of all unigenes (Table 2). There was a relatively large portion of unigenes that had no significant hits to current known proteins, which might represent novel genes in pecan. For functional classification of the assembled unignenes, COG and GO annotation were carried out to gain the distributions of the functional categories (Figure S1).

### 3.2. Analysis of DEGs in the Graft Process of Pecan

Clean reads from the 12 libraries were aligned to the obtained unigenes, and quantified to calculate the expression levels by fragments per kilobase of transcript per million fragments mapped reads (FPKM). Based on the FPKM values of all unigenes, the correlations between biological replicates at each time point were analyzed. We found that there were strong correlations between the biological repetitions, with correlation coefficients over 0.90 (Figure S2).

**Figure 1:**
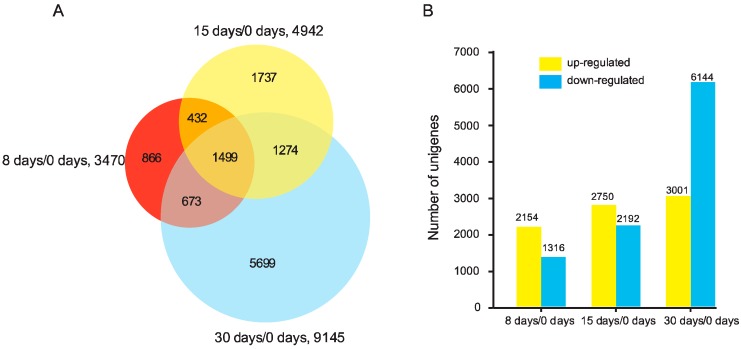
The differentially expressed genes (DEGs) in different comparisons (8 days/0 days, 15 days/0 days, and 30 days/0 days) during graft union development in pecan homografts.

According to the criteria of at least two-fold change and FDR < 0.01, a total of 3470 DEGs were discovered by analyzing 8 days/0 days, with 2154 up-regulated and 1316 down-regulated; 4942 DEGs were identified in the comparison of 15 days/0 days, with 2750 up-regulated and 2192 down-regulated; 9145 DEGs were found by comparing 30 days/0 days, with 3001 up-regulated and 6144 down-regulated. In total, 12,180 DEGs were identified during the grafting process, among which, 1499 genes were detected at all comparisons (Figure 1, Table S3). The number of DEGs in 30 days/0 days was greater than 8 days/0 days and 15 days/0 days, indicating the involvement of complex molecular responses during the developmental stage of vascular tissue formation.

### 3.3. Gene Ontology and Pathway Enrichment Analyses of DEGs

**Figure 2:**
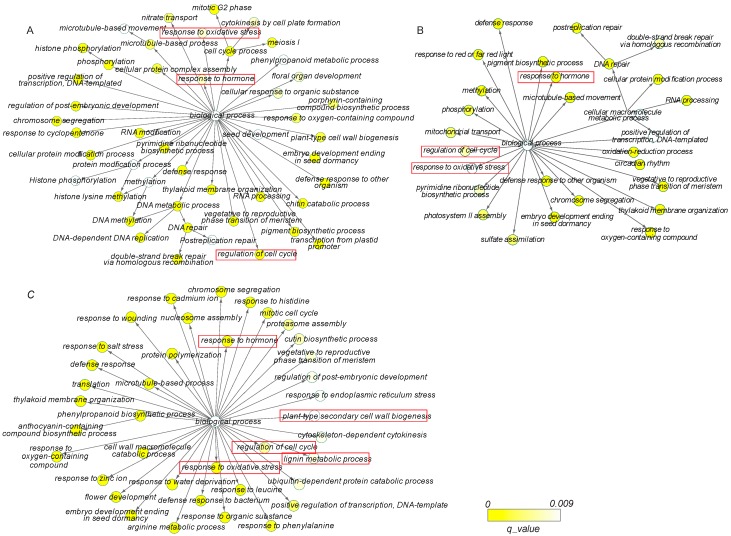
GO enrichment of DEGs during the graft process. (**A**) Significantly enriched GO terms between 8 days and 0 days; (**B**) significantly enriched GO terms between 15 days and 0 days; (**C**) significantly enriched GO terms between 30 days and 0 days. Bubbles represent the significant GO terms, and the bubble color gradient represent the magnitude of enrichment corresponding to *q*-values.

To elucidate the associated biological processes in which the DEGs were involved, the enrichment of GO terms was analyzed. For the ontology of biological process, there were 44, 28, and 38 GO enriched terms in the comparisons of 8 days/0 days, 15 days/0 days, and 30 days/0 days, respectively (Table S4, Figure 2). We found that “response to hormone”, “response to oxidative stress”, and “regulation of cell cycle” were simultaneously enriched in all the comparisons, suggesting the critical roles of these biological processes for a successful grafting. Interestingly, GO terms related to “plant-type secondary cell wall biogenesis” and “lignin metabolic process” were specially enriched in the 30 days vs. 0 days comparison, which were in good agreement with the developmental stage of vasculature formation at 30 days.

**Figure 3:**
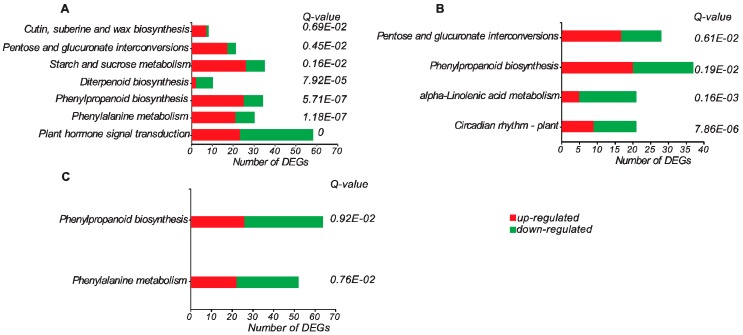
Significant enriched KEGG pathways during the graft process. (**A**) Comparison of 8 days/0 days; (**B**) comparison of 15 days/0 days; (**C**) comparison of 30 days/0 days.

Additionally, KEGG enrichment analysis was performed to reveal the relevant metabolic pathways in which the DEGs participated. We identified 7, 5, and 2 significantly enriched pathways in 8 days/0 days, 15 days/0 days, and 30 days/0 days comparisons, respectively (Figure 3). Among those, the “phenylpropanoid biosynthesis” was the overlapping pathway that was identified in three comparisons, which was consistent with the significant role of this metabolic pathway during the grafting process [10,13].

### 3.4. Hormones Were Critical Regulators for Graft Union Development

**Figure 4:**
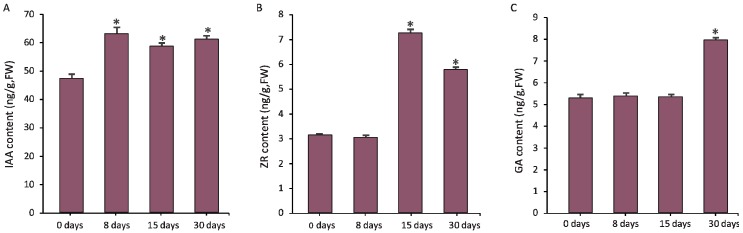
Determination of the contents of hormone at the graft unions during the pecan grafting process. (**A**) Indole acetic acid (IAA); (**B**) zeatin riboside (ZR); and (**C**) gibberellic acid (GA) at different timepoints. * indicates the significant differences (*p* < 0.05) between the specific timepoints and the basal level (0 days).

**Figure 5:**
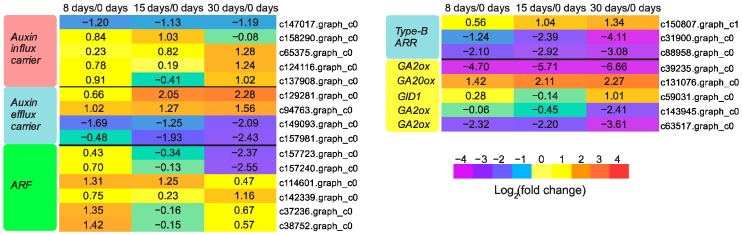
Expression patterns of DEGs involved in hormone signaling. The values of log_2_ fold change are shown in the heat map.

During the grafting process, a block in auxin basipetal transport is produced due to vasculature damage, which leads to auxin accumulation at the graft junction. In our study, the content of auxin was increased distinctly at 8 days, 15 days, and 30 days after grafting (Figure 4A). Correspondingly, all the unigenes except one (c147017.graph_c0) encoding auxin influx carrier, and two unigenes (c129281.graph_c0 and c94763.graph_c0) encoding auxin efflux carrier were significantly up-regulated over the course of graft union development (Figure 5). Similarly, genes responsible for auxin transport were induced during the grafting process of grapevine [6] and hickory [8]. The accumulated auxin is indispensable for the regulation of callus proliferation and cambial activity [14,15]. A high level of auxin would release the transcriptional activity of auxin response factors (ARFs), which would induce the expression of genes that contain auxin responsive elements (AuxREs) in their promoter regions [16,17]. Previous studies have reported that *ARF6* and *ARF8* mutants showed cell division defects [15], and *ARF5* mutants exhibited abnormality in vasculature development [18], indicating auxin signaling via ARFs appeared to be essential for the graft union development. In the present study, six unigenes encoding ARFs were differentially expressed (Figure 5). Intriguingly, three unigenes (c37236.graph_c0, c38752.graph_c0, and c114601.graph_c0) were up-regulated significantly at 8 days or 15 days, indicating a possible role in callus formation; while one unigene (c142339.graph_c0) was greatly up-regulated at 30 days, which suggested it might function in vasculature development.

Additionally, mounting evidence supports the involvement of cytokinin in cell division and vasculature differentiation [19,20,21,22]. Consistent with its role in graft union development, we found that the content of zeatin riboside (ZR), a major form of cytokinin, in graft junction was elevated significantly at 15 days and 30 days (Figure 4B). It has been reported that although auxin is capable of stimulating cell division, cytokinin is required for its full induction [23,24]. Therefore, massive callus proliferation at 15 days in this research might have resulted from the increased cytokinin as well as auxin. Cytokinin signal transduction is mediated via the two-component regulatory pathway to activate type-B ARR transcription factors [25]. Previous studies have showed that triple mutants of *TYPE-B ARRs* (*ARR1*, *ARR10*, *ARR12*) showed reduced callus formation [26], while overexpression of *ARR1* enhanced callus formation [27]. The activated type-B ARRs are likely to be principal regulators of the cytokinin-induced callus proliferation. Three unigenes encoding type-B ARR protein were identified in our DEGs data, and one of them, c150807.graph_c1, was greatly up-regulated at 15 days (Figure 5), which might play an important role in callus formation.

More reports have suggested that gibberellin (GA) could trigger xylogenesis [28,29], which is probably important for the vascular bundle formation cross the grafted partners. This coincided with our biochemical analysis of GA content by ELISA, showing that GA was increased significantly at 30 days, while had no significant difference at other time points (Figure 4C). Accordingly, one GA synthesis gene, *GA20ox*, was highly up-regulated after grafting, and achieved its peak expression at 30 days, while three GA deactivating genes, *GA2ox*, were all significantly down-regulated at 30 days (Figure 5). GA signaling could promote the expression of genes involved in cell expansion, as well as secondary wall biosynthesis during xylem differentiation [30,31]. For the genes involved in GA signaling, we found that one unigene encoding GID1, a gibberellin receptor, was strikingly up-regulated at 30 days, and might take part in vasculature formation.

### 3.5. Genes Responsible for Callus Formation

**Figure 6:**
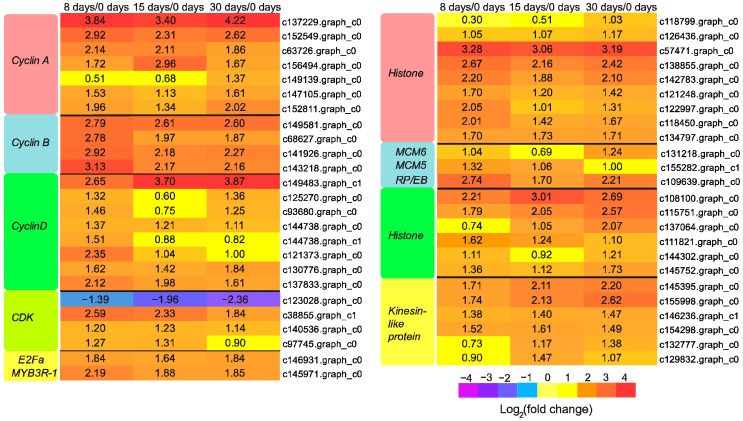
Expression patterns of DEGs involved in and callus formation. The values of log_2_ fold change are shown in the heat map.

Callus formation is a basic wound response to grafting, and the lack of callus production at the graft interface can lead to graft failure [32]. Genes involved in cell division are pivotal for callus formation [33]. Genome-wide transcriptomic study of callus initiation in *Arabidopsis* has revealed the up-regulation of various cell division related genes [34]. Cyclins, together with their catalytically active partners, cyclin dependent kinases (CDKs), are key regulators of cell cycle progression in eukaryotes. Three classes of cyclins (A-, B-, and D-type) exist in plants. Among them, D-type cyclin (CYCD) is a rate limiting factor for the G1/S transition in cell cycle, which plays an important role in driving the entry of cell cycle. It is usually considered as a sensor of external conditions that could be regulated by auxin and cytokinin [20,35,36]. In *Arabidopsis*, overexpression of a *CYCD* led to increased callus growth rate and callus induction frequency [37]. In this work, a considerable number of *cyclins* and *CDKs* were identified and all of them except one, c123028.graph_c0, were up-regulated across the grafting process (Figure 6), which might facilitate the activation of cellular proliferation.

In addition, we found one E2Fa gene, c146931.graph_c0, and one MYB3R-1 gene, c145971.graph_c0, were up-regulated significantly during the entire period of grafting (Figure 6). E2Fa is a transcription factor that drives the expression of genes required for the S-phase in cell cycle [35]. Transgenic *Arabidopsis* overexpressing *E2FA* could induce cell division in tissues already devoid of proliferation [38]. MYB3R-1 is an R1R2R3-type MYB transcription factor that aims at inducing genes required for the M-phase in cell cycle [39]. The up-regulated *E2Fa* along with *MYB3R1* would probably facilitate cell cycle progression in this study. Besides the genes involved in cell cycle, we found that various genes responsible for nucleosome component synthesis (*histone*), DNA replication (*DNA replication licensing factor MCM5* and *MCM6*), microtubule cytoskeleton organization (*microtubule-associated protein RP/EB family* and *tubulin*), and cytokinesis (*kinesin-like protein*) were generally up-regulated over the course of grafting (Figure 6), which might have participated in callus formation.

### 3.6. Genes Participated in Vascular Bundle Formation

**Figure 7:**
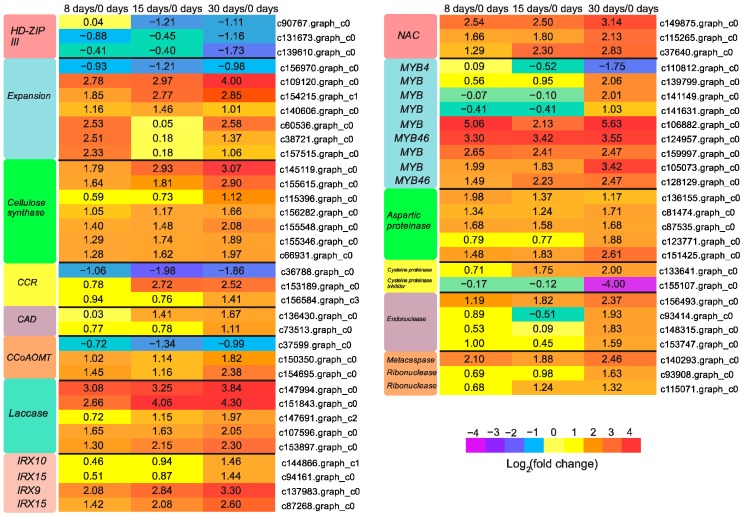
Expression profiles of DEGs involved in vascular bundle formation. Gene expression values were normalized to z-score. The values of log_2_ fold change are shown in the heat map.

Production of new vascular tissues permits the long-distance transport of nutrients between the grafting partners, and is recognized as a mark of successful grafting [32]. Based on our morphological observation of pecan graft union development, xylem is the main vascular tissue at 30 days [11]. The sequential developmental processes underlying wood formation include the promotion of vascular cambial activity, xylem differentiation, cell elongation, secondary cell wall thickening, and programmed cell death [40]. Plant hormones, including auxin and cytokinin, have been implicated in stimulating vascular cambium activity [40]. Previous studies have revealed the involvement of class III homeodomain-leucine zipper (HD-ZIP III) proteins in xylem differentiation [41,42]. It has been reported that the levels of HD-ZIP III determine the identity of xylem. Low levels of HD-ZIP III promote protoxylem identity, while high levels of HD-ZIP III promote metaxylem identity [43]. In our study, three down-regulated *HD-ZIP III* were identified at 30 days (Figure 7), which might function in xylem differentiation. Cells undergo significant enlargement following xylem differentiation. Enzymes such as expansions were required not only for cell growth, but also for the loosening of existing cell wall architecture during cell elongation [44]. As expected, most (six out of seven) unigenes encoding expansion in this study were up-regulated in both the callus proliferative phase and the vasculature formative phase (Figure 7). Tubulin, aside from its role in cell division, also plays a role in cell elongation by guiding nascent cellulose microfibrils deposition [45]. Expression of tubulin genes was also elevated at 30 days in our study (Figure 6). It is presumed that *tubulin* might also be involved in cell expansion during vascular development.

Following the completion of cell elongation, differentiating vascular cells go through the deposition of cellulose, hemicellulose, and lignin in the secondary cell wall. We identified various genes encoding the key biosynthetic enzymes of secondary cell wall components, and most of those genes were up-regulated, with the highest expression value at 30 days (Figure 7). *CELLULOSE SYNTHASE*, a gene implicated in cellulose synthesis, was found to be strongly expressed in the developing secondary xylem of *Populus* [46]. *CINNAMOYL-COA REDUCTASE* (*CCR*), *CINNAMYL-ALCOHOL DEHYDROGENASE* (*CAD*), and *CAFFEOYL COA 3-O-METHYLTRANSFERASE* (*CCoAOMT*) are the genes involved in the phenylpropanoid pathway, all of them critical for monolignol synthesis. The gene product of *LACCASE* is a polyphenol oxidase enzyme, which plays a critical role in lignin formation through inducing the oxidative polymerization of monolignols [47]. Mutations in *LACCASE4* and *17* showed reduced lignin content in *Arabidopsis* [48]. *IRREGULAR XYLEM 9* (*IRX9*), *IRX10*, and *IRX15* are the genes that participate in hemicellulose synthesis.

Additionally, we identified candidate transcription factors involved in the transcriptional regulation of secondary cell wall deposition. Some *NAC* transcription factors are master regulators in controlling the entire developmental process of secondary cell wall synthesis [49]. Overexpression of *NAC* genes in plants induced secondary wall thickening in various tissues, while repression of their function suppressed secondary wall deposition [50,51]. Three particular secondary cell wall related *NACs* were identified, and all the *NACs* were strongly up-regulated at 30 days (Figure 7), which might imply an important role during vasculature differentiation. Secondary wall related *R2R3-type MYB* transcription factors are also important regulators, which have already been identified as transcriptional regulators of phenylpropanoid biosynthesis pathway [52]. In this study, nine candidate *R2R3-type MYB* genes were found to be differentially expressed (Figure 7). Among them, two unigenes, c128129.graph_c0 and c124957.graph_c0, were annotated as *MYB46*, which act as direct targets of NAC domain regulator, exhibiting up-regulation throughout the grafting process with peak value at 30 days (Figure 7). The induced *MYB46* was not only able to activate synthesis of the lignin, but also the cellulose and hemicellulose [53]. One unigene, c110812.graph_c0, was annotated as *MYB4*, a gene that negatively regulated secondary cell wall formation [54], showing great down-regulation at 30 days (Figure 7). Ectopic overexpression of poplar *PdMYB221*, an ortholog of *Arabidopsis MYB4*, was reported to result in decreased thickness of cell walls [55]. Collectively, these DEGs might indicate the synthesis of secondary cell wall components during vasculature differentiation.

After fulfilling secondary cell wall deposition, developing vascular cells trigger programmed cell death (PCD) to digest the cellular contents. Hydrolytic enzymes, such as aspartic proteinase, cysteine proteinase, and nucleases (endonuclease, exonuclease, and ribonuclease) have been demonstrated to operate during xylogenesis [56,57,58], which were generally detected with increased expression at 30 days in our research (Figure 7). Metacaspases are a class of enzymes with structural similarity to animal caspases that could regulate the process of plant programmed cell death [59]. Analysis of microarray data revealed that the expression of an *Arabidopsis METACASPASE 9* (*AtMC9*) homologue gene in *Populus* was up-regulated during xylem maturation [57]. In the present study, the great up-regulation of *metacaspase* at 30 days (Figure 7) might suggest its involvement in vascular bundle differentiation. Otherwise, we found one C*YSTEINE PROTEINASE INHIBITOR* was down-regulated drastically at 30 days (Figure 7), which probably indicated the strong activity of PCD during vascular bundle formation.

### 3.7. Genes Involved in Reactive Oxygen Species (ROS) Scavenging

**Figure 8:**
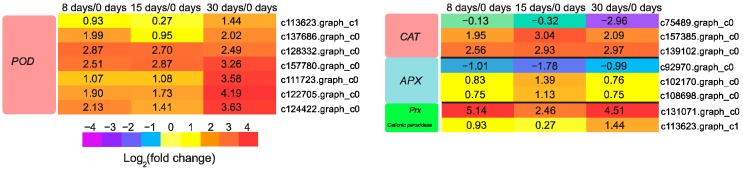
Expression profiles of DEGs involved in ROS scavenging. The values of log_2_ fold change are shown in the heat map.

During grafting, mechanical damage inevitably occurs at the graft interface. In higher plants, the production of ROS is a general event following wounding [60]. There are indications that non-successful grafts show signs of excess oxidative stress [61,62,63]. An efficient antioxidant defense system in plants might be an important factor in achieving successful grafting. In the current investigation, we found 19 DEGs that could scavenge the ROS, including genes encoding peroxidase (POD), catalase (CAT), ascorbate peroxidase (APX), cationic peroxidase, and peroxiredoxin (Prx), and most of them (13 out of 15) showed increased expression during the grafting process (Figure 8), which was presumably related to mitigating the ROS toxicity.

### 3.8. Validation of RNA-Seq Data by Real-Time RT-PCR (qRT-PCR)

**Figure 9:**
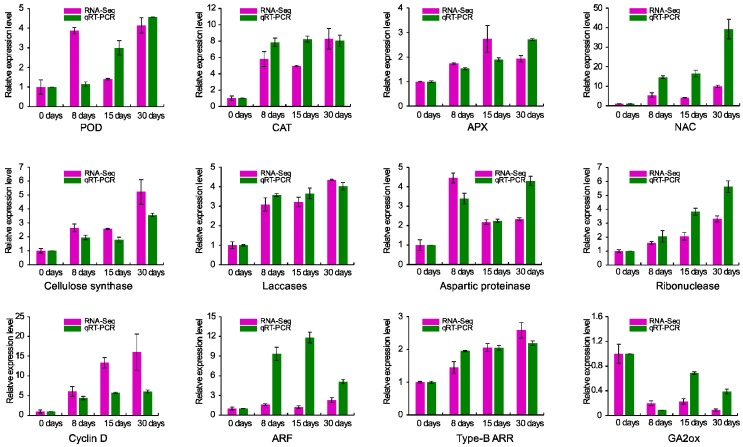
Validation RNA-seq data by real-time quantitative RT-PCR. The expression levels at 0 days are considered as 1.

We selected twelve genes that were predicted to be associated with hormone signaling, cell division, secondary cell wall formation, programmed cell death, and ROS scavenging to validate the sequencing data. Gene expression at different time points was relative to basal level (0 days). For a specific treatment, the fold changes of some genes in their expression detected by qRT-PCR and sequencing did not match exactly, however, the expression patterns of those selected genes were basically identical between the two data sets (Figure 9).

## 4. Conclusions

**Figure 10:**
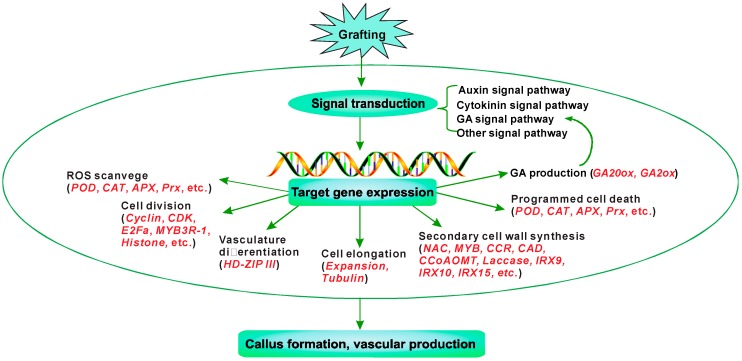
A putative molecular model of successful grafting in pecan.

In this work, transcriptomic analysis was applied to explore the differentially expressed genes at the graft union during the pecan homograft process. A total of 12,180 DEGs were identified at the comparisons of 8 days/0 days, 15 days/0 days, and 30 days/0 days. Candidate genes that would participate in successful grafting were further analyzed. Based on our result, a suggested model for depicting the molecular mechanism of graft union development in pecan could be summarized in Figure 10. Upon grafting, signal transduction pathways including hormone (IAA, CK, and GA) signaling and other unknown signaling are activated. The activated signaling might induce the expression of various genes related to ROS scavenging, cell division, vasculature differentiation, cell elongation, secondary cell wall synthesis, and programmed cell death, resulting in a successful graft.

## Figures and Tables

**Table 1:** Summary for the graft union transcriptome.

	Transcript	Unigene
Total number	140,455	83,693
Total length	165,440,800 bp	74,679,367 np
N50 length	1905 bp	1350 bp
Mean length	1178 bp	892 bp
200–300 bp	18,665 (13.29%)	15,637 (18.68%)
300–500 bp	28,705 (20.44%)	21,661 (25.88%)
500–1000 bp	36,770 (26.18%)	24,333 (29.07%)
1000–2000 bp	31,127 (22.16%)	13,698 (16.37%)
2000+ bp	25,188 (17.93%)	8364 (9.99%)

**Table 2:** Summary for the annotation of unigenes.

Annotated Databases	Unigene Number	Percentage (%)	300 nt ≤ Length < 1000 nt	Length ≥ 1000 nt
COG	11,762	14.05	3747	6644
GO	23,260	27.79	9028	10,879
KEGG	13,859	16.56	5567	6557
KOG	21,612	25.82	8184	10,759
Pfam	25,909	30.96	8838	14,658
Swiss-Prot	23,243	27.77	8711	12,071
NR	38,793	46.35	15,816	17,494
Annotated in at least one database	40,069	47.88	16,463	17,751

## Supplementary Materials

The following are available online at http://www.mdpi.com/2073-4425/9/2/71/s1. Figure S1: Functional categories of assembled unigenes. (A) Distribution of number of annotated unigenes in COG database; (B) Distribution of number of annotated unigenes in GO database; Figure S2: Distribution of correlation co-efficiencies between each pair of samples; Table S1: The primer sequences of selected unigenes; Table S2: Summary of sequences analysis in all samples; Table S3: The identified DEGs in the graft process of pecan; Table S4: Significant enriched GO terms of DEGs between different developmental stages.
