# Supplementary figures and images for: Transcriptomic Analysis Provides Insights into Grafting Union Development in Pecan (Carya illinoinensis)
Source: Genes (Basel). 2018 Feb 1;9(2):71. doi: 10.3390/genes9020071 (PMC5852567; doi:10.3390/genes9020071)

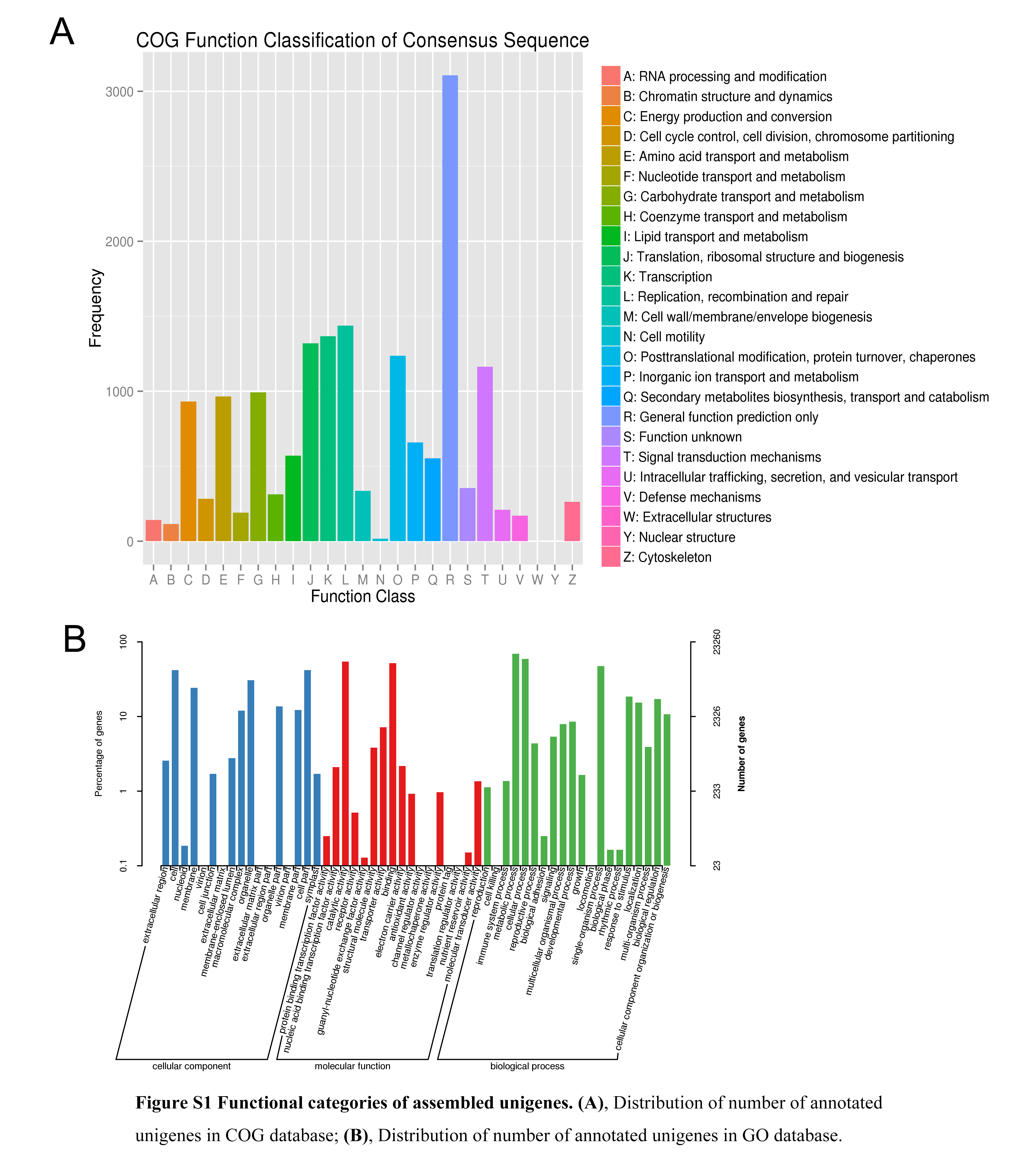

Supplement: Supplementary file 1 [file genes-09-00071-s001.zip › Figure S1 Functional classification of assembled unigenes.tif]

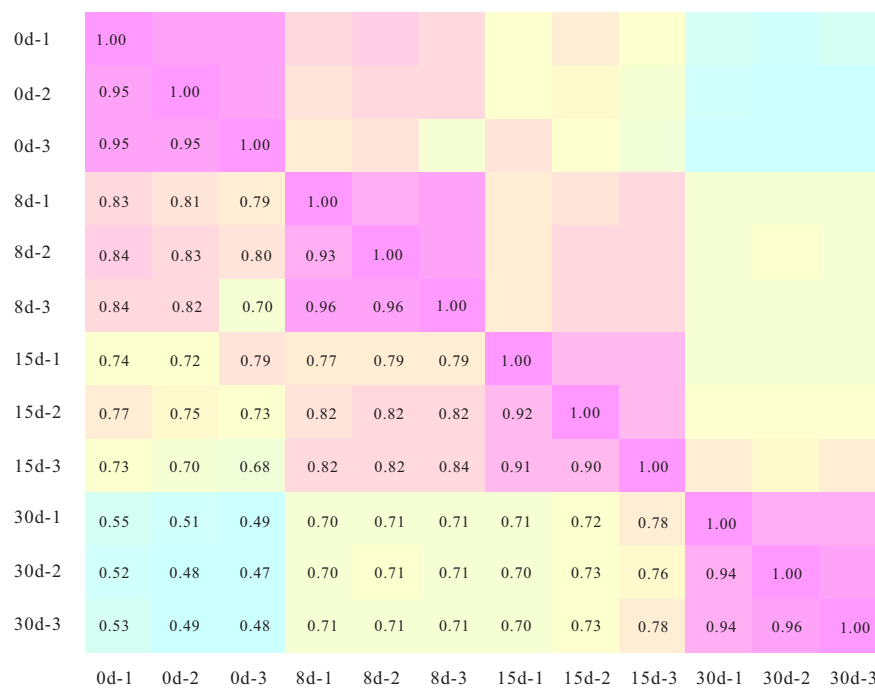

**Figure S2 Distribution of correlation co-efficiencies between each pair of samples**

Supplement: Supplementary file 1 [file genes-09-00071-s001.zip › Figure S2 Distribution of correlation co-efficiencies between each pair of samples.pdf]
